# Supplementary material for: Virus Quasispecies Rarefaction: Subsampling with or without Replacement?
Source: Viruses. 2024 Apr 29;16(5):710. doi: 10.3390/v16050710 (PMC11126021; doi:10.3390/v16050710)
Supplement: Supplementary file 1 [file viruses-16-00710-s001.zip › viruses-2995042-supplementary.pdf]

Quasispecies rarefaction

Subsampling with or without replacement?

Josep Gregori, Marta Ibáñez-Ligoña, Sergi Colomer-Castell
Carolina Campos, and Josep Quer
(josep.gregori@gmail.com, josep.quer@vhir.org)
Liver Diseases - Viral Hepatitis Lab., Vall Hebron Research Institute,
Vall d'Hebron Hospital Campus, Barcelona, Spain

March, 2024

Supplementary material. R code

Subsampling a given fraction with replacement

Theoretical limits to observed items at different subsampling fractions. Table 1 and Figure 1.

```
library(tidyverse)
library(knitr)

### Subsampling a given fraction with replacement
#####################################################

f <- seq(from=0.1,to=1,by=0.1)
rf <- data.frame(Fraction=f,Seen=round(1-exp(-f),4),Unseen=round(exp(-f),4))
rownames(rf) <- NULL
kable(rf,align='c',
      caption=paste(c('Subsampling a given fraction with replacement',
                      'Items seen and unseen in a single resampling cycle.'),
                    collapse=' '))

P <- rf %>%
  ggplot() +
   geom_point(aes(x=Fraction,y=Seen),size=2) +
   geom_abline(intercept=0,slope=1,lty=4,col='grey') +
   geom_hline(yintercept=0.6321,lty=4,col='maroon') +
   geom_vline(xintercept=1,lty=4,col='maroon') +
   geom_text(aes(x=0.20,y=0.66,label=0.6321)) +
   labs(x='Subsampling fraction',y='Observed reads (limit at inf)',
       title='Subsampling with replacement',
       subtitle='Reads sampled') +
   ylim(0.05,1) +
   theme_bw()
print(P)
```

All singletons case

Numeric example about subsampling at different fractions a quasispecies where all reads are unique. Table 2 and function of resampling with replacement used throughout the code.

```
###  All singletons case
#####################################################

###  Quasispecies haplotype distribution
x <- rep(1,10000)

###  Subsampling fractions
f <- seq(from=0.1,to=1,by=0.1)

### Resampling cycles
B <- 500         #****#

###  Diversity function.
###  Number of haplotypes in resample.
div.fn <- function(nr)
  length(nr)


###  Resampling with replacement function  ###
subsample.rep <- function(rds,size,B,div.fn)
{
  one_time <- function()
  { iHpl <- sample.int(length(rds), size=size, replace = TRUE, prob = rds)
    bs.rds <- as.integer(table(iHpl))
    return(div.fn(bs.rds))
  }
  subs.vals <- replicate(B, one_time(), simplify = "array")
  return(subs.vals)
}

###  Compute median, IQR and sd for each fraction
set.seed(151023)
res <- data.frame(Frac=f,Median=0,IQR=0,SD=0)
for(i in 1:length(f))
{ vhpl <-  subsample.rep(x,f[i]*length(x),B,div.fn)
  res$Median[i] <- median(vhpl)
  res$IQR[i] <- IQR(vhpl)
  res$SD[i] <- round(sd(vhpl),2)
}
save(res,file='AllSingl.Rdata')

###  Probability given f and n
sbs <- function(n,f)
```

```
 1-((1-1/n)^(f*n))

res$Expected <- round(sapply(f,function(fr) sbs(length(x),fr)*length(x)),1)
res$True <- length(x)*f
res$Unique <- round(res$Median/(length(x)*f),4)
res$Replicated <- round(1-res$Unique,4)
res <- res %>% select(Frac,True,Expected,Median,IQR,SD,Unique,Replicated)
kable(res,align='ccrrrrcc',
      caption=paste(c('All singleton case.',
                      'Estimating number of haplotypes.',
                      'Subsampling a given fraction with replacement.'),
                    collapse=' '))
```

Single dominant case

Numeric example estimating a prominent frequency and the number of coexisting very rare haplotypes.

Tables 3, 4 and 5, Figures 2 and 3, and function of resampling without replacement used throughout the code.

```
### Single dominant case
####################################################

library(dqrng)

###  Diversity function
###  Number of haplotypes and master frequency
div.fn <- function(nr)
{ frq <- nr/sum(nr)
  c(length(nr),frq[1])
}

###  B subsamples of 'size' reads, without replacement
###    with diversity computations each time.
###  nr: vector of reads per haplotype.
subsample.no.rep <- function(nr,size,B,div.fn)
{ # Mapping of reads to haplotypes
  iHpl <- rep(1:length(nr),times=nr)
  # Sample size
  tnr <- sum(nr)

  # Get subsample and compute diversities
  one_time <- function()
  { # subsample reads
    s <- dqsample.int(tnr,size,replace=FALSE)
```

```
    # get haplotypes indices
    vh <- iHpl[s]
    # vector of haplotype counts
    bs.rds <- as.integer(table(vh))
    # Compute diversity
    return(div.fn(bs.rds))
  }
  # Replicate B times
  subs.vals <- replicate(B, one_time(), simplify = "array")
  return(subs.vals)
}

###  Number of reads in sample
N <- 100000

###  Quasispecies metadata
dict <- data.frame(ID=c('Q.90.10','Q.80.20','Q.70.30','Q.60.40',
                        'Q.50.50','Q.40.60','Q.30.70','Q.20.80',
                        'Q.10.90'),
                   Mstr=seq(from=0.9,to=0.1,by=-0.1))
dict$HplNo <- round(N*(1-dict$Mstr)+1)
kable(dict,align='c',caption='Single dominant, quasispecies data.')

cnms <- c('nHpl','Mstr')

res.df.NoRpl <- data.frame(ID=character(),Subsz=numeric(),q=character(),
                           Median=numeric(),IQR=numeric())
res.df.Rpl <- data.frame(ID=character(),Subsz=numeric(),q=character(),
                         Median=numeric(),IQR=numeric())

set.seed(141023)
for(i in 1:nrow(dict))
{
  rds <- c(N*dict$Mstr[i],rep.int(1,round(N*(1-dict$Mstr[i]))))

  ###  Valors de diversitat per submostreig
  sb <- c(0.5,0.25,0.1,0.05)
  for(j in 1:length(sb))
  { size <- round(sum(rds)*sb[j])
    #cat(i,',',j,',',size,'\n')
    mdv <- t(subsample.no.rep(rds,size,B,div.fn))
    sbs.md.vals <- apply(mdv,2,median)
    names(sbs.md.vals) <- cnms
    sbs.iqr.vals <- apply(mdv,2,IQR)
    names(sbs.iqr.vals) <- cnms
    res.df.NoRpl <- rbind(res.df.NoRpl,
      data.frame(ID=dict$ID[i],Subsz=sb[j],q=cnms,Median=sbs.md.vals,
                 IQR=sbs.iqr.vals))
```

```
    mdv <- t(subsample.rep(rds,size,B,div.fn))
    sbs.md.vals <- apply(mdv,2,median)
    names(sbs.md.vals) <- cnms
    sbs.iqr.vals <- apply(mdv,2,IQR)
    names(sbs.iqr.vals) <- cnms
    res.df.Rpl <- rbind(res.df.Rpl,
      data.frame(ID=dict$ID[i],Subsz=sb[j],q=cnms,Median=sbs.md.vals,
                 IQR=sbs.iqr.vals))
  }
}
save(res.df.NoRpl,res.df.Rpl,file='SinglDom.RData')

### Sampling without replacement
SngDom.NoRpl <- NoRpl <- res.df.NoRpl %>%
  select(-IQR) %>%
  pivot_wider(names_from='q',values_from='Median') %>%
  data.frame()

### Sampling with replacement
SngDom.Rpl <- Rpl <- res.df.Rpl %>%
  select(-IQR) %>%
  pivot_wider(names_from='q',values_from='Median') %>%
  data.frame()

###  Combined table. Estimating number of haplotypes
SngDom.HplEst <- NoRpl %>%
  select(ID,Subsz,nHpl) %>%
  dplyr::rename(NoRpl=nHpl) %>%
  mutate(WithRpl=Rpl$nHpl) %>%
  left_join(dict %>% select(ID,HplNo),by='ID') %>%
  mutate(Exact=round(HplNo*Subsz)) %>%
  select(-HplNo)
kable(SngDom.HplEst,align='ccrrr',
      caption=paste(c('Single dominant.', 'Estimating number of haplotypes.',
                      'Median values.'),collapse=' ' ))

###  Single dominant case. Number of haplotypes plot
P <- SngDom.HplEst %>%
  mutate(Subsz=factor(Subsz)) %>%
  mutate(NoRpl=NoRpl/Exact,WithRpl=WithRpl/Exact) %>%
  select(-Exact) %>%
  pivot_longer(-c(ID,Subsz),names_to='Type',values_to='HplNo') %>%
  ggplot() +
    geom_col(aes(x=ID,y=HplNo,fill=Type),position='dodge',
             col='navy',lwd=0.2) +
    facet_grid(~Subsz) +
    labs(x='',y='Fraction of true haplotypes',title='Single dominant case') +
```

```
    theme_bw() +
    theme(legend.position='bottom') +
    theme(axis.text.x = element_text(angle = 90, vjust = 0.5, hjust=1))
print(P)

###  Combined table. Estimating master frequency
SngDom.MstrEst <- NoRpl %>%
  select(ID,Subsz,Mstr) %>%
  dplyr::rename(NoRpl=Mstr) %>%
  mutate(WithRpl=Rpl$Mstr) %>%
  left_join(dict %>% select(ID,Mstr),by='ID') %>%
  dplyr::rename(Exact=Mstr)
kable(SngDom.MstrEst,align='c',
      caption=paste(c('Single dominant.', 'Estimating master frequency.',
                      'Median values.'),collapse=' ' ))

###  Single dominant case. Master frequency plot
P <- SngDom.MstrEst %>%
  mutate(Subsz=factor(Subsz)) %>%
  mutate(NoRpl=NoRpl/Exact,WithRpl=WithRpl/Exact) %>%
  select(-Exact) %>%
  pivot_longer(-c(ID,Subsz),names_to='Type',values_to='Master') %>%
  ggplot() +
    geom_col(aes(x=ID,y=Master,fill=Type),position='dodge',
             col='navy',lwd=0.2) +
    facet_grid(~Subsz) +
    labs(x='',y='Fraction of true master freq.',title='Single dominant case') +
    theme_bw() +
    theme(legend.position='bottom') +
    theme(axis.text.x = element_text(angle = 90, vjust = 0.5, hjust=1))
print(P)
```

Prominent haplotypes

Numeric example about a quasispecies with six prominent haplotypes and coexisting very rare haplotypes.

Tables 6 and 7.

```
### Prominent haplotypes
####################################################

### Quasispecies featuring six prominent haplotypes
N <- 100000
fr <- c(1,1/2,1/4,1/8,1/16,1/32,1/16)
frn <- fr/sum(fr)
```

```
n <- round(frn *N)
nr <- c(n[1:6],rep(1,n[7]))

{ cat("  Number of sequences:",sprintf("%6d",N),"\n")
  cat("Number of haplotypes:",length(nr),'\n')
  cat("Prominent haplotypes:",sprintf('%5d',nr[1:6]),'\n')
  cat("                     ",round(nr[1:6]/sum(nr),3),'\n')
  cat("          Singletons:",sum(nr==1),'\n')
}
true.df <- data.frame('True',matrix(0,1,8))
colnames(true.df) <- c('Subs','SngFr',paste('Hpl',1:6,sep='_'),'Ov1')
true.df[1,2:9] <- c(frn[7],frn[1:6],0.)

###  Diversity function
div.fn <- function(nr)
{ tnr <- sum(nr)
  ###  Fraction of singletons
  sng <- sum(nr==1)/tnr
  ###  Top 6 haplotypes
  nr <- sort(nr,decreasing=TRUE)
  top6 <- nr[1:6]/tnr
  nr <- nr[-(1:6)]
  ###  Fraction over 1 read, below the top 6
  ov1 <- sum(nr[nr>1])/tnr
  c(sng,top6,ov1)
}

###  Subsampling fractions
sb <- c(0.5,0.25,0.1,0.05)
m <- length(sb)

cnms <- c('SngFr',paste('Hpl',1:6,sep='_'),'Ov1')
res.NoRpl <- matrix(0,m,length(cnms))
colnames(res.NoRpl) <- cnms

cnms <- c('SngFr',paste('Hpl',1:6,sep='_'),'Ov1')
res.Rpl <- matrix(0,m,length(cnms))
colnames(res.Rpl) <- cnms

for(j in 1:length(sb))
{ size <- round(sum(nr)*sb[j])
  mdv <- t(subsample.no.rep(nr,size,B,div.fn))
  sbs.md.vals <- apply(mdv,2,median)
  res.NoRpl[j,] <- sbs.md.vals

  mdv <- t(subsample.rep(nr,size,B,div.fn))
  sbs.md.vals <- apply(mdv,2,median)
  res.Rpl[j,] <- sbs.md.vals
```

```
}
save(res.NoRpl,res.Rpl,file='PromHpl.RData')


###  Subsampling without replacement, table of diversity values
res.df.NoRpl <- data.frame(Subs=c(paste(sb)),res.NoRpl)
res.df.NoRpl <- rbind(true.df,res.df.NoRpl)
res.df.NoRpl <- res.df.NoRpl %>% mutate(across(where(is.numeric),~round(.,5)))
kable(res.df.NoRpl,align='c',
      caption=paste(c('Prominent haplotypes.',
                      'Subsampling without replacement.',
                      'Median values.'),collapse=' '))

###  Subsampling with replacement, table of diversity values
res.df.Rpl <- data.frame(Subs=c(paste(sb)),res.Rpl)
res.df.Rpl <- rbind(true.df,res.df.Rpl)
res.df.Rpl <- res.df.Rpl %>% mutate(across(where(is.numeric),~round(.,5)))
kable(res.df.Rpl,align='c',
      caption=paste(c('Prominent haplotypes.',
                      'Subsampling with replacement.',
                      'Median values.'),collapse=' '))
```

No rare haplotypes

Numeric example of a qusispecies with no rare haplotypes.

Tables 8 and 9.

```
### No rare haplotypes
####################################################

N <- 100000
fr <- c(0.9,rep(0.01,10))
frn <- fr/sum(fr)
nr <- round(frn*N)
cat("  Number f sequences:",sprintf("%d",N),"\n")
cat("Number of haplotypes:",length(nr),'\n')

true.df <- data.frame('True',matrix(0,1,12))
colnames(true.df) <-
   c('Subs','HplNo',paste('Hpl',sprintf("%02d",1:11),sep='_'))
true.df[1,2:13] <- c(length(nr),frn)

###  Diversity function
div.fn <- function(nr)
{ tnr <- sum(nr)
```

```
  ###  Haplotypes
  hpl <- length(nr)
  ###  Frequencies
  frq <- nr/tnr
  frq <- c(frq,rep(0,11-length(frq)))
  c(hpl,frq)
}

###  Subsampling fractions
sb <- c(0.5,0.25,0.1,0.05)
m <- length(sb)

cnms <- c('HplNo',paste('Hpl',sprintf("%02d",1:11),sep='_'))
res.NoRpl <- matrix(0,m,length(cnms))
colnames(res.NoRpl) <- cnms

res.Rpl <- matrix(0,m,length(cnms))
colnames(res.Rpl) <- cnms

for(j in 1:length(sb))
{ size <- round(sum(nr)*sb[j])
  mdv <- t(subsample.no.rep(nr,size,B,div.fn))
  sbs.md.vals <- apply(mdv,2,median)
  res.NoRpl[j,] <- sbs.md.vals

  mdv <- t(subsample.rep(nr,size,B,div.fn))
  sbs.md.vals <- apply(mdv,2,median)
  res.Rpl[j,] <- sbs.md.vals
}
save(res.Rpl,res.NoRpl,file='NoRareHpl.RData')

###  Subsampling without replacement. Table of values.
res.df.NoRpl <- data.frame(Subs=c(paste(sb)),res.NoRpl)
res.df.NoRpl <- rbind(true.df,res.df.NoRpl)
kable(res.df.NoRpl, align='c',
      caption=paste(c('No rare haplotypes.',
                      'Subsampling without replacement'),collapse=' '))

###  Subsampling with replacement. Table of values.
res.df.Rpl <- data.frame(Subs=c(paste(sb)),res.Rpl)
res.df.Rpl <- rbind(true.df,res.df.Rpl)
kable(res.df.Rpl, align='c',
      caption=paste(c('No rare haplotypes.',
                      'Subsampling with replacement'),collapse=' '))
```

Flat quasispecies

Numeric example about a highly diverse quasispecies, whith a high number of haplotypes, where all have identical frequencies.

Table 10 and Figures 4, 5, and 6.

```
### Flat quasispecies
#####################################################

###  Number of haplotypes
n <- 10000
###  Growing multiplicity
k <- 1:10

###  Bootstrap probability given n and m
emp <- function(n,k)
  1-(1-k/(k*n))^(k*n)

###  Bootstrap limit as n goes to infinity
lim <- function(k)
  1-(1/exp(1))^k

res <- data.frame(nHpl=n,k=1:10,Reads=n*k,Prob=emp(n,k),Limit=lim(k))
kable(res,align='r',caption='Flat quasispecies, growing frequencies')

P <- res %>%
  ggplot() +
  geom_line(aes(x=k,y=Limit)) +
  geom_point(aes(x=k,y=Limit),size=2) +
  labs(x='Reads per haplotype',y='Fraction of haplotypes sampled',
       title='Flat quasispecies bootstrap') +
  scale_x_continuous(breaks=1:10, labels=1:10) +
  theme_bw()
print(P)

#------------------------------------------------------------#

###  Subsampling a given fraction f with replacement

n <- 10000
k <- 1:10
f <- seq (0.1,1,0.1)

### Limit as n goes to infinity
lim <- function(k,f)
1-(1/exp(1))^(k*f)

calc.f <- matrix(0,nrow=length(k),ncol=length(f))
rownames(calc.f) <- sprintf("k%02d",k)
```

```
colnames(calc.f) <- sprintf("f%.2f",f)

for(i in 1:length(k))
  for(j in 1:length(f))
    calc.f[i,j] <- lim(k[i],f[j])

###  Estimating number of haplotypes from the rarefaction equation

raref <- function(N,k,f)
  N - exp(log(N)+lchoose(N*k-k,round(N*k*f))-lchoose(N*k,round(N*k*f)))

res.raref <- matrix(0,nrow=length(k),ncol=length(f))
rownames(res.raref) <- sprintf("k%02d",k)
colnames(res.raref) <- sprintf("f%.2f",f)

for(i in 1:length(k))
  for(j in 1:length(f))
    res.raref[i,j] <- raref(n,k[i],f[j])

raref.df <- res.raref %>% data.frame() %>%
  rownames_to_column(var='k') %>%
  pivot_longer(-k,names_to='f',values_to='FrSeen')  %>%
  mutate(k=substring(k,2),f=substring(f,2)) %>%
  mutate(FrSeen=FrSeen/N)

lb.df <- raref.df %>% filter(f=='0.10')

P <- raref.df %>%
  ggplot() +
   geom_line(aes(x=f,y=FrSeen,group=k,col=k),lwd=0.8) +
   # geom_abline(aes(intercept=0,slope=0.1),lwd=0.8,col='grey',lty=4) +
   geom_text(aes(x=0.75,y=FrSeen,label=as.integer(k)),data=lb.df) +
   geom_text(aes(x=0.75,y=0.7,label='k')) +
   labs(x="Subsampling fraction",y="Fraction of haplotypes observed",
        col="Reads\nper\nHpl.",title="Flat quasispecies rarefaction",
        subtitle='Rarefaction equation') +
   theme_bw(base_size=12)

pdf("FlatQs.Rarefaction.pdf",width=5.5,height=5)
print(P)
dev.off()

ratio <- round(calc.f/(res.raref/N),4)

P <- ratio %>% data.frame() %>%
  rownames_to_column(var='k') %>%
  pivot_longer(-k,names_to='f',values_to='Ratio')  %>%
  mutate(k=substring(k,2),f=substring(f,2)) %>%
```

```
  ggplot() +
   geom_line(aes(x=f,y=Ratio,group=k,col=k),lwd=0.8) +
   labs(x="Subsampling fraction",y="Fraction of haplotypes observed",
        col="Reads\nper\nHpl.",title="Flat quasispecies. ",
        subtitle='Replacement vs. Rarefaction') +
   theme_bw(base_size=12)

pdf("FlatQs.ReplacementVsRarefaction.pdf",width=5.5,height=5)
print(P)
dev.off()
```

Session info

```
R version 4.2.2 (2022-10-31 ucrt) Platform: x86_64-w64-mingw32/x64 (64-bit)
Running under: Windows 10 x64 (build 22621)
Matrix products: default

locale: [1] LC_COLLATE=Catalan_Spain.utf8 LC_CTYPE=Catalan_Spain.utf8
[3] LC_MONETARY=Catalan_Spain.utf8  LC_NUMERIC=C LC_TIME=Catalan_Spain.utf8

attached  base packages:
[1] stats graphics grDevices utils datasets methods base

other attached packages:
[1]         dqrng_0.3.1 knitr_1.44 lubridate_1.9.2 forcats_1.0.0 stringr_1.5.0
[6] dplyr_1.1.2 purrr_1.0.1 readr_2.1.4 tidyr_1.3.0 tibble_3.2.1
[11] ggplot2_3.4.2 tidyverse_2.0.0

loaded via a namespace (and not attached):
[1] Rcpp_1.0.10 bslib_0.5.1 compiler_4.2.2 pillar_1.9.0 jquerylib_0.1.4
[6] tools_4.2.2 digest_0.6.31 timechange_0.2.0 jsonlite_1.8.7 evaluate_0.22
[11] lifecycle_1.0.3 gtable_0.3.3 pkgconfig_2.0.3 rlang_1.1.1
[15] cli_3.6.1 rstudioapi_0.14 yaml_2.3.7 xfun_0.39 fastmap_1.1.1
[20] withr_2.5.0 hms_1.1.3 generics_0.1.3 sass_0.4.7 vctrs_0.6.2
[25] grid_4.2.2 tidyselect_1.2.0 glue_1.6.2 R6_2.5.1 fansi_1.0.4
[30] rmarkdown_2.25.1 bookdown_0.34 farver_2.1.1 tzdb_0.4.0 magrittr_2.0.3
[35] scales_1.2.1 htmltools_0.5.5 colorspace_2.1-0 labeling_0.4.2 utf8_1.2.3
[40] stringi_1.7.12 munsell_0.5.0 cachem_1.0.8
```
